# Hypnosis and relaxation techniques in the management of temporomandibular disorders: a scoping review

**DOI:** 10.22514/jofph.2026.048

**Published:** 2026-07-12

**Authors:** Estelle Casazza, Benoit Ballester, Maxime Joncour, Yamina Benamara-Tlemsani, Jean-Philippe Ré, Anne Giraudeau

**Affiliations:** ^1^Aix Marseille Univ, APHM, CNRS, EFS, ADES, Hôpital de la Timone, Pôle PROMOD ODONTO, Service d’Odontologie Hospitalière et Chirurgie orale, 13005 Marseille, France; ^2^Aix Marseille Univ, APHM, Inserm, IRD, SESSTIM, Sciences Economiques & Sociales de la Santé & Traitement de l’Information Médicale, ISSPAM, Hôpital de la Timone, Pôle PROMOD ODONTO, Service de Réhabilitations Orales, 13005 Marseille, France; ^3^Aix Marseille Univ, APHM, Hôpital de la Timone, Pôle PROMOD ODONTO, Service d’Odontologie Hospitalière et Chirurgie orale, 13005 Marseille, France

**Keywords:** Temporomandibular disorders, Hypnosis, Self-hypnosis, Relaxation training, Pain, Biopsychosocial model

## Abstract

Temporomandibular disorders (TMD) are common causes of orofacial pain, influenced by psychological and behavioural factors. Non-pharmacological interventions, such as hypnosis and relaxation techniques, have been proposed to modulate pain perception, reduce muscle hyperactivity, and improve coping. However, their role in TMD management remains unclear. We conducted a scoping review to synthesize the available evidence on the use of hypnosis and relaxation interventions in adolescents and adults with TMD. A systematic search of PubMed, Embase, Web of Science, Cochrane Library, PsycINFO, and Google Scholar databases from January 1992 to January 2026 identified a limited number of eligible studies (n = 10), comprising randomized controlled trials and quasi-experimental studies, with substantial methodological heterogeneity in study design. Methodological quality was appraised using a simplified Joanna Briggs Institute tool. Interventions included medical hypnosis, hypnosis combined with cognitive-behavioural therapy, and various relaxation techniques, delivered as stand-alone or adjunctive therapies. Findings for the 661 participants were inconsistent: hypnosis-based interventions suggested potential reductions in pain intensity and psychological distress, whereas relaxation techniques showed mixed results, particularly when compared to standard treatments such as occlusal splints. Methodological appraisal revealed variability in study quality, with three studies at low risk of bias, four at moderate risk, and three at high risk. Overall, the small number of studies and their marked heterogeneity substantially limit the interpretability and comparability of findings, thereby constraining their integration into a robust evidence-based biopsychosocial model for TMD management. Consequently, current evidence remains preliminary and insufficient to support firm clinical recommendations. Nevertheless, these approaches are conceptually aligned with the biopsychosocial model of TMD, and preliminary results suggest that hypnosis and relaxation may represent low-risk adjunctive strategies within multimodal TMD management, particularly for patients with stress-sensitive or centrally sensitized pain profiles. Future well-designed, adequately powered trials with standardized interventions and multidimensional outcomes are needed to clarify their clinical utility.

## 1. Introduction

Temporomandibular disorders (TMD) have been defined as “a heterogeneous group of conditions affecting the temporomandibular joints (TMJ), the jaw muscles, and the related structures” [1]. They represent one of the most common causes of non-dental orofacial pain and may affect approximately 30% of the global population, with a higher prevalence in women [2, 3]. The main clinical manifestations include orofacial pain, limitation of mandibular movement, mandibular deviation, and joint noise, all of which may substantially impair patients’ quality of life [4, 5].

The current understanding of TMD is based on a biopsychosocial model, involving complex interactions between biomechanical, neuromuscular, psychosocial, and behavioural factors [6, 7]. A growing body of evidence highlights the central role of psychological factors (such as stress, anxiety, and depression) in the onset, persistence, and chronification of TMD [8, 9, 10, 11]. This complexity has led to recommendations that favour multimodal, conservative, and patient-centred therapeutic approaches that integrate physical, educational, and psychological interventions [12, 13, 14, 15]. Accordingly, non-pharmacological strategies aimed at modulating stress, pain perception, and emotional responses have attracted increasing attention [16].

Hypnosis, defined as “a state of consciousness involving focused attention and reduced peripheral awareness characterized by an enhanced capacity for response to suggestion” [17], and relaxation therapy, which aims to induce a passive mode of thinking by focusing attention on some neutral target, such as a body part or breathing [18, 19] are consistent with this biopsychosocial perspective. These approaches seek to modulate pain perception, reduce muscle hyperactivity, enhance coping skills, and promote patient self-management [20]. They are already used in the management of other chronic pain conditions, including musculoskeletal pain such as low back pain and cancer-related pain, with variable but generally encouraging results [21, 22]. However, their use in the management of TMD remains less clearly defined [18]. Indeed, available studies differ substantially in terms of study populations, intervention protocols, and outcome measures, thereby hindering data synthesis and the formulation of clear clinical recommendations [23].

The objective of this scoping review was to synthesize the available evidence on hypnosis and relaxation techniques in the management of temporomandibular disorders and to identify areas of weakness to inform future research.

## 2. Materials and methods

This review was carried out in accordance with the Preferred Reporting Items for Systematic Reviews and Meta-Analyses extension for Scoping Reviews (PRISMA-ScR) protocol (**Supplementary Table 1**) [24].

### 2.1 Research question and eligibility criteria

This scoping review was conducted in line with the PCC (Population-Concept-Context) framework [25] (Table 1).

**Table 1.** Development of the research question using the PCC framework.

Population	Concept	Context
Studies conducted in populations of adolescents and adults diagnosed with temporomandibular disorders (TMD) were considered eligible. No restrictions were applied regarding gender, duration of symptoms, or diagnostic criteria, provided that a TMD was clinically identified.	Studies investigating hypnosis and/or relaxation techniques as therapeutic interventions were included. These techniques encompassed clinical hypnosis, self-hypnosis, progressive muscle relaxation, guided relaxation, and related approaches.	Studies conducted in any clinical or healthcare setting were considered, provided that the interventions were delivered within the context of conservative or multimodal management of TMD.

The review posed the research question. “What evidence is available regarding the use of hypnosis and relaxation techniques in the management of temporomandibular disorders?”.

#### 2.1.1 Inclusion criteria

Studies were eligible if they included adolescents (>12 years old) or adults diagnosed with temporomandibular disorders and investigated the use of hypnosis and/or relaxation techniques as therapeutic interventions, either as stand-alone treatments or within a multimodal management approach. All study designs reporting clinical, functional, or psychosocial outcomes were considered. Studies had to be approved by an ethics committee and be written in English. Articles published between January 1992 (the year of publication of the Research Diagnostic Criteria for Temporomandibular Disorders—RDC/TMD) and January 2026 were included.

#### 2.1.2 Exclusion criteria

Studies focusing exclusively on non-TMD orofacial pain conditions, paediatric populations, purely pharmacological interventions, or psychological interventions without a defined hypnosis or relaxation component were excluded. Editorials, expert opinions, and animal studies were also excluded.

### 2.2 Data collection

The scientific article search was conducted in six online databases—PubMed, Embase, Web of Science, Cochrane Library, PsycINFO, and Google Scholar—from January 1992 to 15 January 2026 by two blinded operators. When possible, database-specific date filters were applied, while the custom range function was used for Google Scholar. Studies were excluded based on titles and abstracts and after reading the full articles. All phases of the review process (study selection and data extraction) were independently assessed by two evaluators (EC and BB) using the collaborative tool Revstack [26]. In cases of disagreement, the reviewers discussed the studies in detail and reached consensus based on predefined eligibility criteria and standardized data extraction procedures. When necessary, the original articles were re-examined to ensure accurate classification and data reporting. All discrepancies were resolved through discussion between the two evaluators.

The search strategy combined terms related to temporomandibular disorders with terms related to hypnosis and relaxation techniques. Reference lists of included studies were manually screened to identify additional relevant publications.

#### 2.2.1 Search equation for PubMed

((“Temporomandibular Disorders” [Mesh] OR “temporomandibular disorder*” [Title/Abstract] OR “TMD” [Title/Abstract] OR “myofascial pain” [Title/Abstract]) AND (“Hypnosis” [Mesh] OR hypno* [Title/Abstract] OR “relaxation therapy” [Mesh] OR “relaxation” [Title/Abstract] OR “progressive muscle relaxation” [Title/Abstract] OR “autogenic training” [Title/Abstract])) AND (“1992/01/01” [Date-Publication]: “2026/01/15” [Date-Publication]).

#### 2.2.2 Search equation for Web of Science

TS = ((“temporomandibular disorder*” OR “TMD” OR “myofascial pain”) AND (“hypno*” OR “relaxation therapy” OR “progressive muscle relaxation” OR “autogenic training”)).

Timespan: 1992–2026.

TS = Topic.

#### 2.2.3 Search equation for Embase

(“temporomandibular joint disorder”/exp OR “temporomandibular disorder*” OR “myofascial pain”/exp) AND (“hypnosis”/exp OR hypno* OR “relaxation therapy”/exp OR “progressive muscle relaxation” OR “autogenic training”) AND [1992–2026]/py.

#### 2.2.4 Search equation for Cochrane Library

(“temporomandibular disorder*” OR “TMD” OR “TMJ” OR “orofacial pain” OR “jaw pain”) AND ((“hypnosis” OR “hypnotherapy” OR “clinical hypnosis”) OR (“relaxation” OR “relaxation therapy” OR “progressive muscle relaxation” OR “autogenic training”)).

#### 2.2.5 Search equation for PsycINFO

((“temporomandibular disorder*” OR “TMJ” OR “TMD” OR “orofacial pain” OR “jaw pain” OR “craniomandibular disorder*”)) AND ((hypnosis OR hypnotherapy OR “clinical hypnosis”) OR (“relaxation therapy” OR relaxation OR “progressive muscle relaxation” OR “autogenic training”)).

(PY ≥1992 AND PY ≤2026).

PY = Publication Year.

#### 2.2.6 Search equation for Google Scholar

(“temporomandibular disorder” OR “TMD” OR “TMJ” OR “orofacial pain”) AND (hypnosis OR hypnotherapy OR relaxation OR “relaxation therapy”) filetype: pdf.

Given the low specificity and the ranking algorithm of the Google Scholar database, only the first 100 records sorted by relevance were screened for eligibility.

### 2.3 Qualitative analysis of results

A qualitative descriptive synthesis was produced using the PCC tool (Population-Concept-Context) to map study characteristics, intervention protocols, and reported outcomes.

Methodological quality was assessed using a simplified version of the Joanna Briggs Institute (JBI) critical appraisal tools, with checklists adapted to study design (randomized controlled trials and quasi-experimental studies) [27, 28]. Given the scoping nature of this review, our appraisal was conducted to describe methodological trends and potential sources of bias rather than to exclude studies or to compute pooled quality scores. The inclusion of heterogeneous study designs, while appropriate for a scoping review, increased methodological variability, limiting cross-study comparability and weakening the strength of inferences, thus reflecting a trade-off between breadth of evidence mapping and depth of causal inference.

The following key domains were considered: design, groups comparable at baseline, adequacy of randomization, allocation concealment, blinding procedures, attrition, outcome measure reliability, and confounding factors. Attrition was considered methodologically acceptable when loss to follow-up did not exceed 15% of the initial sample. Studies exceeding this threshold were judged to be at increased risk of bias due to potentially incomplete outcome data.

Overall risk of bias (low, moderate, or high) was determined using a transparent, domain-based approach without numerical scoring. Studies were classified as low risk when no key domain was judged to be at high risk of bias and only minor concerns were identified. Risk was assessed as moderate when one or more domains raised concerns or were rated as unclear, without being considered critical to internal validity. Studies were assessed as carrying a high risk of bias when at least one domain was judged to be critically flawed, for example, inadequate randomization, substantial attrition (>15%), or unreliable outcome measurement, or when multiple domains were at risk. Greater weight was given to domains considered central to internal validity (*e.g.*, randomization, completeness of outcome data, and measurement reliability).

## 3. Results

### 3.1 Study selection

The study search strategy is shown in a flow chart (Fig. 1, Ref. [29, 30, 31, 32, 33, 34, 35, 36, 37, 38]). A total of 720 records were identified through database searching (PubMed, Embase, Web of Science, Cochrane Library, PsycINFO, and Google Scholar). After removal of 182 duplicates, 538 records were screened by title and abstract, and 519 of these records were excluded. Sixteen full-text articles were assessed for eligibility, and ten studies met the inclusion criteria and were included in the qualitative synthesis. The most common reason for exclusion at the full-text stage was the absence of a clearly defined hypnosis or relaxation intervention.

**Fig. 1.** PRISMA-ScR flow diagram of the search for studies on the use of hypnosis and relaxation techniques in the management of temporomandibular disorders [29, 30, 31, 32, 33, 34, 35, 36, 37, 38]. TMD: Temporomandibular disorders.

### 3.2 Extraction of data

The characteristics of each study identified by the PCC analysis are shown in Table 2 (Ref. [29, 30, 31, 32, 33, 34, 35, 36, 37, 38]).

**Table 2.** Study characteristics and description of intervention.

Study	Study design	Population (total N, average age, gender, TMD type, diagnostic criteria)	Group description (intervention and comparator)	Concept (Type of intervention)	Delivery mode (group, individual, mixed, self-administered)	Dose (number of sessions, duration, frequency)	Context
Oakley, 1994 [29]	QES	56 adult patients, mean age 35 years 85% women Mixed TMD Clinical examination	G1: relaxation (n = 32) G2: no treatment (n = 24)	G1: Guided relaxation and self-hypnosis at home	Group Self-administered (with audiotapes)	6 sessions; 1.5 hours; once weekly; 6 weeks total	Stand-alone (after unsuccessful standard treatment), monocentric
Simon, 2000 [30]	QES	28 adult patients, mean age 33 years 89% women Mixed TMD Clinical examination	G1: hypnosis (n = 28) No control group	G1: Hypnosis and self-hypnosis at home	Group Self-administered (with audiotapes)	6 sessions; once weekly; 6 weeks total	Stand-alone (after unsuccessful standard treatment), monocentric
Winocur, 2002 [31]	RCT	40 adult patients, mean age 30 years Women-only TMD muscular subgroup RDC/TMD	G1: hypnosis, relaxation, and self-hypnosis (n = 15) G2: OA (n = 15) G3: MT (n = 10)	G1: Hypnosis, relaxation, and self-hypnosis at home	No details regarding patient distribution (individual *vs.* group sessions) Self-administered (using audiotapes)	5 sessions; once weekly; 5 weeks total	Stand-alone, monocentric
Wahlund, 2003 [32]	RCT	122 adolescent patients, mean age 15 ± 2 years, 76% girls Mixed TMD RDC/TMD	G1: BI and relaxation (n = 41) G2: BI and OA (n = 42) G3: BI (n = 39)	G1: Relaxation and daily home practice	Individual	4 sessions; every 2 weeks; 8 weeks total	Stand-alone, monocentric
Abrahamsen, 2009 [33]	RCT	40 adult patients, mean age 38.6 ± 10.8 years, women-only Mixed TMD RDC/TMD	G1: hypnosis (n = 20) G2: relaxation (n = 20)	G1: Hypnosis	Individual	4 sessions; 1 hour each; 5 weeks total	Stand-alone, monocentric
Abrahamsen, 2011 [34]	RCT	43 adult patients, mean age 38.6 years ± 10.9 Women-only TMD muscular subgroup RDC/TMD	G1: hypnosis and self-hypnosis (n = 21) G2: relaxation (n = 22)	G1: Hypnosis Self-hypnosis at home	Individual Self-administered (using compact disc)	4 sessions; 1 hour each; 5 weeks total	Stand-alone, monocentric
Ferrando, 2012 [35]	RCT	72 adult patients, mean age 39 years 90% women TMD muscular subgroup RDC/TMD	G1: CBT and hypnosis with standard treatment (OA, jaw exercises, medication) (n = 41) G2: Standard treatment (OA, jaw exercises, medication) (n = 31)	G1: Cognitive-Behavioural Technique and hypnosis	No details regarding patient distribution (individual *vs.* group sessions)	6 sessions; 1 hour each; 2.5 months	Adjunctive therapy, monocentric
Wahlund, 2015 [36]	RCT (2 phases)	64 adolescent patients 16.4 years ± 1.87 95% girls Mixed TMD RDC/TMD	Phase 1 G1: Relaxation (n = 31) G2: OA (n = 33) Phase 2 G1: n = 8 G2: n = 23	G1: Relaxation and daily home practice	Individual Self-administered (manual and audiotapes)	8 sessions; 45 minutes; 8 weeks	Stand-alone, monocentric
Ferendiuk, 2019 [37]	QES	100 adult patients TMD muscular subgroup RDC/TMD	G1: Relaxation (Jacobson protocol) (n = 50) G2: post-isometric muscle relaxation (n = 50)	G1: Relaxation (Jacobson protocol)	No details regarding patient distribution (individual *vs.* group sessions)	5 sessions; 45 minutes; twice a week; 2.5 weeks total	Stand-alone, monocentric
Huhtela, 2020 [38]	RCT	96 adult patients, 25 years 85% women Mixed TMD RDC/TMD	G1: relaxation (n = 55) G2: OA (n = 41)	G1: Applied Relaxation (protocol by Öst modified by Thorsel)	No details regarding patient distribution (individual *vs.* group sessions)	6 sessions; 8 weeks total	Stand-alone, Monocentric

*TMD: temporomandibular disorder; QES: Quasi-Experimental Study; RCT: Randomized Controlled Trial; RDC/TMD: Research Diagnostic Criteria for Temporomandibular Disorders; OA: Occlusal Appliance; MT: Minimal Treatment; BI: Brief Information; CBT: Cognitive-Behavioural Therapy.*

Main outcomes and results are presented in Table 3 (Ref. [29, 30, 31, 32, 33, 34, 35, 36, 37, 38]). Principal quantitative pain intensity data are reported when available; otherwise, results are summarized narratively with an indication of statistical significance.

**Table 3.** Main outcomes and results of included studies.

Study	Main Outcomes	Time points (baseline, post-intervention, follow-up)	Main results and statistically significant effects (* or NS)
Oakley, 1994 [29]	Pain Intensity (VAS) Quality, intensity, and pattern of pain (McGill Pain Questionnaire) Anxiety (Spielberger’s State-Trait Anxiety Inventory) Depression (Beck Depression Inventory)	Baseline, post-intervention	Moderate effect on pain (NS) Reduction of psychological distress: G1 > G2*
Simon, 2000 [30]	Pain (TMD questionnaires) Medical use	Baseline, post-intervention 6-month follow-up	Pain reduction* Medical use reduction* Gain maintained during follow-up*
Winocur, 2002 [31]	Pain (VAS) Depression and somatization (RDC/TMD self-questionnaires)	Baseline, post-intervention	Pain reduction G1: −3.5 VAS G2: −3 VAS G3: −0.6 G1 > G3* G1 = G2 (NS) No information on depression or somatization
Wahlund, 2003 [32]	Pain (VAS) Analgesic consumption School absence	Baseline, post-intervention 6-month follow-up	Pain reduction: G1: −1 VAS G2: −2 VAS G3: −0.5 VAS G2 > G1 (NS) G2 > G3* Analgesic consumption: G2 > G3* School absence: G1 > G2 > G3 (NS) Gains maintained during follow-up
Abrahamsen, 2009 [33]	Pain (NRS) Psychological symptoms (Symptom Check List 60) Pain coping strategies (Coping Strategies Questionnaire) Sleep difficulties (Pittsburgh Sleep Quality Index) Analgesic consumption	Baseline, post-intervention	Pain reduction: G1: −1.5 NRS G2: −0.3 NRS G1 > G2* Reduction of psychological distress in both groups*
Abrahamsen, 2011 [34]	Pain (NRS)	Baseline, post-intervention	Pain reduction G1: −1.5 NRS G2: −0.5 NRS G1 > G2*
Ferrando, 2012 [35]	Pain (McGill Pain Questionnaire, Multidimensional Pain Inventory) Somatization, depression, and anxiety (Brief Symptoms Inventory)	Baseline, post-intervention 9-month follow-up	Pain reduction G1 > G2* Reduction of somatization, depression, and anxiety G1 > G2* Gains maintained during follow-up
Wahlund, 2015 [36]	Pain (VAS) Analgesic consumption School absence	Baseline, post-intervention 6-month follow-up	Pain reduction: Phase 1: G1: −1 NRS G2: −2 NRS G2 > G1* Phase 2: G1: 0 NRS G2: −0.5 NRS G2 = G1 (NS) Analgesic consumption: G1 = G2 (NS) School absence: G1 = G2 (NS) Gain maintained during follow-up
Ferendiuk, 2019 [37]	Pain (RDC/TMD questionnaire)	Baseline, post-intervention	Pain reduction G1 > G2*
Huhtela, 2020 [38]	Pain (VAS) Depressive and non‐specific physical symptoms (RDC/TMD self-questionnaire)	Baseline, post-intervention 12-month follow-up	Pain reduction G1: −1 NRS G2: −1 NRS G1 = G2 (NS) Reduction of psychological distress G1 > G2* Gain maintained during follow-up

VAS: Visual Analogic Scale; NRS: Numeric Rating Scale; RDC/TMD: Research Diagnostic Criteria for Temporomandibular Disorders; NS: No statistically significant difference. *Statistically significant difference.

### 3.3 Synthesis of results

#### 3.3.1 Geographic distribution

The articles included in the final step of the review represented a broad geographical distribution, including Sweden (n = 2), Finland (n = 1), Denmark (n = 2), Poland (n = 1), Spain (n = 1), the United States of America (n = 2) and Israel (n = 1).

#### 3.3.2 Sample characteristics

A total of 661 participants were enrolled in the selected studies, including 475 adults and 186 adolescents. A marked predominance of female participants was observed in nearly all samples, and in three studies the participants were exclusively women [31, 33, 34].

#### 3.3.3 Diagnostic criteria and TMD subtype

Except for the two earliest studies, TMD was diagnosed using the RDC/TMD criteria [29, 30]. The questionnaires used differed between studies, but the same markers were sought: limitation of mouth opening and pain on palpation for Axis I, and depression, anxiety, and somatization for Axis II. Most participants presented with muscular TMD (masticatory muscle disorders), although some studies covered both articular and muscular TMD, which were designated as “mixed TMD” in Table 2. No study differentiated clearly between articular subtypes in outcome analyses.

#### 3.3.4 Intervention characteristics

Ten studies were included in our review, comprising seven randomized controlled trials and three quasi-experimental studies. All were monocentric. Populations were predominantly adult and female, with two studies focusing on adolescents. Study samples consisted of either mixed TMD subtypes or muscular TMD subtypes only, with diagnoses based on clinical examination or RDC/TMD criteria.

Interventions included hypnosis, relaxation techniques, or their combination, sometimes integrated with cognitive-behavioural therapy. Delivery formats varied substantially, and included individual sessions, group-based interventions, and self-administered home practice supported by audio or written materials. Treatment duration ranged from 2.5 to 8 weeks (4–8 sessions). Most interventions were delivered as stand-alone treatments, except for one study, which evaluated a multimodal approach involving a combination of cognitive-behavioural therapy and hypnosis [35].

#### 3.3.5 Reported outcomes

The primary outcome in all studies was pain intensity, assessed with a VAS (Visual Analogue Scale) [29, 31, 32, 38], NRS (Numeric Rating Scale) [33, 34, 36], or validated questionnaires [30, 35, 37]. Psychological outcomes (anxiety, depression, somatization) and functional measures (analgesic use, school absence, healthcare utilization) were also frequently reported. Assessments were typically conducted at baseline and post-intervention, with follow-up (ranging from 6 to 12 months) available in five studies.

The outcome measures employed were heterogeneous, which complicated inter-study comparisons.

#### 3.3.6 Principal results

Overall, hypnosis and relaxation interventions were associated with improvements in pain-related and psychological outcomes, although results were heterogeneous. Pain reduction was observed in most studies, with significant between-group differences in favour of the intervention groups in several trials [33, 34, 35, 37], although this was not consistent [29, 38]. In some studies, comparable effects were observed in both the intervention and occlusal appliance groups [31, 38].

In contrast, psychological outcomes showed more consistent improvement, with significant reductions in anxiety, depression, or somatization reported in multiple studies, including those in which pain reduction was not statistically significant. The combination of hypnosis with cognitive-behavioural therapy yielded the most consistent benefits in both pain-related and psychological domains.

Age-related differences emerged, as occlusal appliances were generally more effective than relaxation-based interventions in adolescent populations [32, 36], whereas comparable effects were observed for these two approaches in adults [31, 35].

#### 3.3.7 Follow-up assessment

A number of studies investigated the effects of treatments over several months [30, 32, 35, 36, 38]. Follow-up data suggested that treatment effects, particularly for pain and psychological outcomes, may be maintained for 6 to 12 months. However, the limited number of studies and the heterogeneity of interventions and outcomes preclude firm conclusions regarding long-term effectiveness.

### 3.4 Methodological evaluation

The methodological quality of the included studies was assessed using the Joanna Briggs Institute (JBI) critical appraisal tools for randomized controlled trials (RCTs) and quasi-experimental studies (QES). The results of this appraisal are shown in Table 4 (Ref. [29, 30, 31, 32, 33, 34, 35, 36, 37, 38]).

**Table 4.** Methodological appraisal (JBI-based synthesis).

Study	Design	Groups comparable at baseline	Randomi-zation	Allocation concealment	Blinding	Attrition	Outcome reliability	Confoun-ding factors	Overall risk of bias
Oakley, 1994 [29]	QES	Unclear	N/A	N/A	Unclear	N/A	Yes	Unclear	High risk
Simon, 2000 [30]	QES	N/A (no control group)	N/A	N/A	No	No	Yes	Unclear	High risk
Winocur, 2002 [31]	RCT	Yes	Yes	Yes	Yes	Yes	Yes	Yes	Low risk
Wahlund, 2003 [32]	RCT	Yes	Yes	Unclear	No	Yes	Yes	Yes	Moderate risk
Abrahamsen, 2009 [33]	RCT	Yes	Yes	Yes	Yes	Yes	Yes	Yes	Low risk
Abrahamsen, 2011 [34]	RCT	Yes	Yes	Yes	Yes	Yes	Yes	Yes	Low risk
Ferrando, 2012 [35]	RCT	Yes	Yes	Yes	Yes	No	Yes	Yes	Moderate risk
Wahlund, 2015 [36]	RCT	Yes	Yes	Unclear	No	Yes	Yes	Yes	Moderate risk
Ferendiuk, 2019 [37]	QES	Unclear	N/A	N/A	No	N/A	Yes	Unclear	High risk
Huhtela, 2020 [38]	RCT	Yes	Yes	Yes	No	No	Yes	Yes	Moderate risk

*N/A: not applicable; QES: Quasi-Experimental Study; RCT: Randomized Controlled Trial. Unclear: insufficient information reported.*

Overall methodological quality across the seven RCTs was generally moderate to high. Most studies reported adequate randomization procedures, though in some, the allocation concealment procedure was unclear [32, 36] or blinding was incomplete due to the nature of the intervention [32, 36, 38]. Attrition levels were mostly acceptable, except for the studies of Ferrando *et al*. [35] and Huhtela *et al*. [38], which reported participant dropout levels that may have introduced bias. All the RCTs reviewed used validated outcome measures, using validated pain and psychological assessment tools. Confounding factors were generally well controlled through randomization, though baseline differences were noted in a few studies. Overall, three RCTs were judged to be at low risk of bias [31, 33, 34], while four were rated at moderate risk due to unclear allocation concealment or incomplete blinding [32, 35, 36, 38].

The three QES were assessed to be at high risk of bias across multiple domains. One did not include a control group [30], while baseline comparability was unclear in others [29, 37]. Randomization and allocation concealment were not applicable, and blinding was either unclear or absent. Reporting of attrition was inconsistent, and confounding factors were generally not addressed. Outcome reliability was sometimes reported, but often without validated tools. These limitations reflect the inherent bias of single-group or non-randomized designs.

Our risk of bias assessment highlights the fact that evidence from RCTs is more robust, whereas quasi-experimental studies provide exploratory or preliminary data only. Notably, more recent trials demonstrated greater methodological rigour than earlier studies, suggesting a progressive strengthening of research standards in this field. Studies with a high risk of bias were not excluded but were interpreted with caution.

## 4. Discussion

This scoping review, which focuses on the value of hypnosis and relaxation in the management of patients with TMD, identified ten scientific articles published in the international literature. These studies presented heterogeneous populations (adults and adolescents), methodologies, intervention protocols, and outcome measures. Some of the included studies suggested potential effects on reduced orofacial pain intensity following hypnosis or relaxation-based interventions. Our methodological appraisal was intended to contextualize findings rather than to determine comparative effectiveness. The diversity of methodology and the limited number of high-quality randomized controlled trials preclude firm conclusions regarding clinical efficacy. In light of the limited and preliminary nature of the evidence, hypnosis and relaxation cannot currently be considered evidence-based primary treatments and should instead be viewed as adjunctive options.

The place of hypnosis and relaxation in the therapeutic arsenal remains marginal, even though these techniques have been the subject of research for several decades. However, as shown by Jogna *et al*. [16], the main TMD therapies described by studies between 2014 and 2024 were occlusal appliance and manual therapy; hypnosis and relaxation were among the least-represented care modalities. Nevertheless, the clinical rationale for their use in TMD management is consistent with current concepts of chronic orofacial pain. Indeed, our contemporary understanding of TMD is predicated on a biopsychosocial model, as reflected in the Diagnostic Criteria for Temporomandibular Disorders (DC/TMD), which represents a further development of the RDC/TMD [5]. This model emphasizes the dynamic interaction between biological factors (*e.g.*, muscle dysfunction, joint pathology), psychological processes (*e.g.*, anxiety, catastrophizing, hypervigilance), and social determinants [39]. Psychological distress, stress-related muscle hyperactivity, maladaptive coping strategies, and sleep disturbances have all been implicated in the onset and maintenance of chronic TMD pain [40]. In line with this biopsychosocial model, recent clinical practice guidelines for the management of chronic pain associated with temporomandibular disorders provide strong recommendations in favour of cognitive behavioural therapy, with or without relaxation therapy, highlighting the relevance of interventions that target the psychological and behavioural dimension of pain [13]. In this context, hypnosis and relaxation training may exert their therapeutic effects by targeting central mechanisms involved in pain modulation and affective regulation [41, 42]. More specifically, functional neuroimaging studies indicate that hypnosis can alter activity in the anterior cingulate cortex, the insula, and prefrontal regions, which are key nodes in the affective and sensory dimensions of pain [43, 44]. Relaxation training may reduce sympathetic overactivity, attenuate masticatory muscle hypertonicity, and normalize stress-related neuroendocrine responses. These effects are particularly relevant in TMD, where central sensitization and hypervigilance contribute to symptom chronicity and disability [45].

Most of the included studies evaluated changes in pain intensity, typically using visual analogue scales [29, 31, 32, 38] or numerical rating scales [33, 34, 36]. In some trials, improvements were comparable to those observed with standard conservative treatments, such as occlusal splints or manual therapy [31, 35, 38]. However, sample sizes were frequently small, limiting statistical power. Moreover, follow-up periods, present in five studies [30, 32, 35, 36, 38], were fairly long (several months). In addition, in two studies, adolescent patients [32, 36] were followed up over a period of several years (average age: 14 years) [46], and long-term maintenance of benefit was encouraging, despite the number of patients who discontinued participation. So, patient adherence emerged as a critical determinant of effectiveness. The difficulties involved in setting up the studies, which were spread over several weeks and required a high level of involvement on the part of participants, were one of the reasons for the small number of studies available on the subject. Interventions that incorporate self-guided elements, home practice, or digital support may enhance engagement and facilitate long-term maintenance of benefits.

Beyond pain intensity, all studies assessed secondary outcomes, including anxiety levels, depressive symptoms, and sleep quality, assessed using a range of questionnaires. Although not uniformly reported, improvements in psychological distress and coping were observed in most studies. These findings are clinically relevant, as emotional distress and maladaptive cognitions such as catastrophizing are recognized as contributing to pain chronicity and disability. The observed reductions in anxiety and stress are consistent with the known effects of both hypnosis and relaxation training on autonomic regulation and emotional processing [47]. Such changes may not only influence pain perception directly but also improve adherence to other therapeutic modalities. Thus, even in cases where direct analgesic effects are modest, the broader psychological impact may justify integrating these approaches into comprehensive TMD management [48].

Methodological appraisal using the simplified JBI tool highlighted variability in study quality. Although several randomized controlled trials were identified, common limitations included small sample sizes, insufficient reporting of allocation concealment, limited blinding procedures, and heterogeneous outcome measures. Non-randomized studies frequently lacked control groups [29, 30] and reported incomplete follow-up data [37], which restricts the interpretability and generalizability of findings. Moreover, high attrition rates may result in the overestimation of treatment effects and reflect challenges in long-term adherence to behavioural interventions. In addition, considerable heterogeneity was observed in intervention protocols, including differences in session number, duration, therapeutic scripts, delivery mode (individual *vs.* self-guided), and practitioner expertise. Indeed, some studies employed individualized hypnotic suggestions targeting pain modulation [33, 34], whereas others used standardized scripts or self-hypnosis, using a compact disc or recorded tape at home [30, 31, 33]. Similarly, relaxation training ranged from progressive muscle relaxation [37] to multimodal stress management programmes [29, 32, 38]. This variability complicated cross-study comparisons and limited the identification of specific active components.

Although methodological differences existed between studies, a certain degree of homogeneity was found in TMD diagnosis, due to the use of the RDC/TMD. This method, proposed in 1992, is based on a dual assessment of TMD: Axis I for physical diagnoses and Axis II for diagnosis of psychological status and pain-related disability. This evaluation aims to simultaneously provide a physical diagnosis and identify other relevant patient characteristics that could influence the expression and thus the management of TMD. Two studies did not mention this diagnostic method [29, 30]; however, they were included in the analysis as they were based on a clinical examination and psychological status questionnaires similar to those described in the RDC/TMD. Importantly, some studies clearly distinguished between muscular and articular disorders, and all included patients with masticatory muscle disorders, which is plausible, as interventions targeted stress and muscle tension. However, the inclusion of heterogeneous TMD subtypes should be acknowledged as a potential source of variability in treatment response. Indeed, muscular TMD are more directly linked to psychosocial factors, parafunctional habits, and increased muscle activity, which may make them particularly responsive to interventions such as hypnosis and relaxation. In contrast, articular TMD, which often involve structural or inflammatory joint alterations, may respond to these approaches differently or to a lesser extent. This heterogeneity, although understandable given the limited number of studies available, may have influenced the outcomes observed and limits the comparability of results across studies.

### 4.1 Clinical implications

The findings of this review support the consideration of hypnosis and relaxation training techniques as adjunctive components of multimodal TMD management. They are generally low-risk, non-invasive interventions with minimal adverse effects when delivered by trained practitioners. They may be particularly relevant for patients presenting with high levels of anxiety, exacerbation of stress-related symptoms, or chronic pain unresponsive to conventional conservative treatments. However, these approaches should not be viewed as stand-alone alternatives to established evidence-based treatments. Instead, they may complement occlusal therapy, physiotherapy, patient education, and cognitive behavioural strategies, within an individualized care plan consistent with the biopsychosocial model [23].

Age-related differences should also be considered. TMD management is not currently well validated in children and adolescents, and non-invasive, reversible approaches should be preferred in these populations [49]. Some studies suggest that hypnosis and relaxation techniques may be less effective in adolescents than occlusal splint therapy. Given that the aetiology, clinical presentation, and treatment response of TMD may vary between adolescents and adults, these interventions may require adaptation according to patient age.

### 4.2 Limitations

The authors chose to conduct a scoping review to identify the types of evidence available in the research field on the use of hypnosis and relaxation techniques in the management of patients with TMD. The main limitation of this review lies in its search strategy. Indeed, this may have prevented the identification of all studies of interest because of database coverage limitations and the particularities of article indexing.

The possibility of comparing the results of these studies was therefore limited owing to the small number of articles available and their considerable methodological disparity. For this reason, it was not possible to carry out a meta-analysis based on this review.

### 4.3 Future research prospects

Future research should prioritize well-designed, adequately powered multicentre randomized trials with clearly standardized and well-reported hypnosis and relaxation protocols to improve reproducibility and comparability. Studies should adopt a biopsychosocial framework by incorporating multidimensional outcomes extending beyond pain intensity, to include psychological distress, coping, disability, and quality of life. Mechanistic investigations targeting central pain modulation and autonomic regulation are also needed. Finally, better patient stratification may help identify those TMD subgroups most likely to benefit from these interventions within a multimodal management approach.

## 5. Conclusion

Hypnosis and relaxation techniques represent conceptually coherent, low-risk, and non-invasive adjuncts for the management of temporomandibular disorders within a biopsychosocial model. The limited evidence available suggests potential effects on pain reduction, stress-related muscle hyperactivity, and emotional regulation, which are key mechanisms implicated in TMD chronicity. However, these findings remain preliminary due to the small number of studies, intervention heterogeneity, and variability in intervention methodologies. While these approaches could be considered as elements in individualized, multimodal treatment strategies, particularly for patients with stress-sensitive or centrally sensitized TMD phenotypes, robust conclusions regarding their clinical effectiveness cannot yet be drawn. Future well-designed, adequately powered trials incorporating standardized intervention protocols and multidimensional outcome measures are needed to clarify their clinical role and to identify those patient subgroups most likely to benefit. Advancing research in this area may help expand integrative, mechanism-based approaches to TMD management consistent with contemporary biopsychosocial models of orofacial pain.

## Supplementary Material

Supplementary material associated with this article can be found, in the online version, at https://files.jofph.com/files/article/2075421085547806720/attachment/Supplementary%20material.pdf.



## References

[1] Manfredini D, Häggman-Henrikson B, Al Jaghsi A, Baad-Hansen L, Beecroft E, Bijelic T, *et al*. Temporomandibular disorders: INfORM/IADR key points for good clinical practice based on standard of care. CRANIO®. 2025; 43: 1–5. 10.1080/08869634.2024.2405298 39360749

[2] Valesan LF, Da-Cas CD, Réus JC, Denardin ACS, Garanhani RR, Bonotto D, *et al*. Prevalence of temporomandibular joint disorders: a systematic review and meta-analysis. Clinical Oral Investigations. 2021; 25: 441–453. 10.1007/s00784-020-03710-w 33409693

[3] Zieliński G, Pająk-Zielińska B, Ginszt M. A meta-analysis of the global prevalence of temporomandibular disorders. Journal of Clinical Medicine. 2024; 13: 1365. 10.3390/jcm13051365 PMC10931584 38592227

[4] Fillingim RB, Slade GD, Greenspan JD, Dubner R, Maixner W, Bair E, *et al*. Long-term changes in biopsychosocial characteristics related to temporomandibular disorder: findings from the OPPERA study. Pain. 2018; 159: 2403–2413. 10.1097/j.pain.0000000000001348 PMC6193833 30028791

[5] Schiffman E, Ohrbach R, Truelove E, Look J, Anderson G, Goulet JP, *et al*. Diagnostic criteria for temporomandibular disorders (DC/TMD) for clinical and research applications: recommendations of the International RDC/TMD Consortium Network* and Orofacial Pain Special Interest Group^†^. Journal of Oral & Facial Pain and Headache. 2014; 28: 6–27. 10.11607/jop.1151 PMC4478082 24482784

[6] Slade GD, Fillingim RB, Sanders AE, Bair E, Greenspan JD, Ohrbach R, *et al*. Summary of findings from the OPPERA prospective cohort study of incidence of first-onset temporomandibular disorder: implications and future directions. Journal of Pain. 2013; 14: T116–T124. 10.1016/j.jpain.2013.09.010 PMC3857103 24275219

[7] Schiffman E, Ohrbach R. Executive summary of the diagnostic criteria for temporomandibular disorders for clinical and research applications. Journal of the American Dental Association. 2016; 147: 438–445. 10.1016/j.adaj.2016.01.007 PMC4884471 26922248

[8] Fillingim RB, Ohrbach R, Greenspan JD, Knott C, Diatchenko L, Dubner R, *et al*. Psychological factors associated with development of TMD: the OPPERA prospective cohort study. Journal of Pain. 2013; 14: T75–T90. 10.1016/j.jpain.2013.06.009 PMC3855656 24275225

[9] Canales GDLT, Guarda-Nardini L, Rizzatti-Barbosa CM, Conti PCR, Manfredini D. Distribution of depression, somatization and pain-related impairment in patients with chronic temporomandibular disorders. Journal of Applied Oral Science. 2019; 27: e20180210. 10.1590/1678-7757-2018-0210 PMC6322638 30624469

[10] Reis PHF, Laxe LAC, Lacerda-Santos R, Münchow EA. Distribution of anxiety and depression among different subtypes of temporomandibular disorder: a systematic review and meta-analysis. Journal of Oral Rehabilitation. 2022; 49: 754–767. 10.1111/joor.13331 35398904

[11] Felin GC, Tagliari CV da C, Agostini BA, Collares K. Prevalence of psychological disorders in patients with temporomandibular disorders: a systematic review and meta-analysis. Journal of Prosthetic Dentistry. 2024; 132: 392–401. 10.1016/j.prosdent.2022.08.002 36114016

[12] Al-Moraissi EA, Conti PCR, Alyahya A, Alkebsi K, Elsharkawy A, Christidis N. The hierarchy of different treatments for myogenous temporomandibular disorders: a systematic review and network meta-analysis of randomized clinical trials. Oral and Maxillofacial Surgery. 2022; 26: 519–533. 10.1007/s10006-021-01009-y 34674093

[13] Busse JW, Casassus R, Carrasco-Labra A, Durham J, Mock D, Zakrzewska JM, *et al*. Management of chronic pain associated with temporomandibular disorders: a clinical practice guideline. The BMJ. 2023; 383: e076227. 10.1136/bmj-2023-076227 38101929

[14] Ferrillo M, Nucci L, Giudice A, Calafiore D, Marotta N, Minervini G, *et al*. Efficacy of conservative approaches on pain relief in patients with temporomandibular joint disorders: a systematic review with network meta-analysis. CRANIO®. 2025; 43: 258–274. 10.1080/08869634.2022.2126079 36148997

[15] Bijelic T, Michelotti A, Bucci R, Del Sorbo D, Ekberg E, Häggman-Henrikson B. Self-management therapies for temporomandibular disorders—evidence from systematic reviews. Journal of Oral Rehabilitation. 2026; 53: 265–281. 10.1111/joor.70074 PMC12705301 41058307

[16] Jogna F, Graenicher AA, Rey-Millet Q, Groz A, De Grasset J, Stollar F, *et al*. Pharmacological and non-pharmacological approaches to temporomandibular disorder chronic pain: a narrative review. Pain Management. 2025; 15: 285–296. 10.1080/17581869.2025.2502311 PMC12118397 40338653

[17] Elkins GR, Barabasz AF, Council JR, Spiegel D. Advancing research and practice: the revised APA division 30 definition of hypnosis. American Journal of Clinical Hypnosis. 2015; 57: 378–385. 10.1080/00029157.2015.1011465 25928776

[18] Benson H, Beary JF, Carol MP. The relaxation response. Psychiatry. 1974; 37: 37–46. 10.1080/00332747.1974.11023785 4810622

[19] Zhang Y, Montoya L, Ebrahim S, Busse JW, Couban R, McCabe RE, *et al*. Hypnosis/Relaxation therapy for temporomandibular disorders: a systematic review and meta-analysis of randomized controlled trials. Journal of Oral & Facial Pain and Headache. 2015; 29: 115–125. 10.11607/ofph.1330 25905529

[20] Phillips W, Price J, Molyneux PD, Deeley Q. Hypnosis. Practical Neurology. 2022; 22: 42–47. 10.1136/practneurol-2020-002839 34389642

[21] Wortzel J, Spiegel D. Hypnosis in cancer care. American Journal of Clinical Hypnosis. 2017; 60: 4–17. 10.1080/00029157.2017.1290577 28557681

[22] Langlois P, Perrochon A, David R, Rainville P, Wood C, Vanhaudenhuyse A, *et al*. Hypnosis to manage musculoskeletal and neuropathic chronic pain: a systematic review and meta-analysis. Neuroscience & Biobehavioral Reviews. 2022; 135: 104591. 10.1016/j.neubiorev.2022.104591 35192910

[23] Silva FWD, Oliveira DV, Zina LG, Paula JS. Use of hypnosis in the treatment of orofacial pain: a systematic review. Journal of Integrative and Complementary Medicine. 2025; 31: 224–232. 10.1089/jicm.2024.0154 39365879

[24] Tricco AC, Lillie E, Zarin W, O’Brien KK, Colquhoun H, Levac D, *et al*. PRISMA extension for scoping reviews (PRISMA-ScR): checklist and explanation. Annals of Internal Medicine. 2018; 169: 467–473. 10.7326/M18-0850 30178033

[25] Descatha A, Morin E, Pitet S, Fadel M, Mayet A, Huard-Dutertre L, *et al*. Scoping reviews and umbrella reviews: principle, methodology, and practice in occupational health. Archives of Occupational and Environmental Diseases. 2025; 86: 102946. (In French)

[26] Ballester B. Revstack. 2025. Available at: https://revstack.gitbook.io/docs (Accessed: 24 February 2026).

[27] Barker TH, Stone JC, Sears K, Klugar M, Tufanaru C, Leonardi-Bee J, *et al*. The revised JBI critical appraisal tool for the assessment of risk of bias for randomized controlled trials. JBI Evidence Synthesis. 2023; 21: 494–506. 10.11124/JBIES-22-00430 36727247

[28] Barker TH, Habibi N, Aromataris E, Stone JC, Leonardi-Bee J, Sears K, *et al*. The revised JBI critical appraisal tool for the assessment of risk of bias quasi-experimental studies. JBI Evidence Synthesis. 2024; 22: 378–388. 10.11124/JBIES-23-00268 38287725

[29] Oakley ME, McCreary CP, Clark GT, Holston S, Glover D, Kashima K. A cognitive-behavioral approach to temporomandibular dysfunction treatment failures: a controlled comparison. Journal of Orofacial Pain. 1994; 8: 397–401. 7670428

[30] Simon EP, Lewis DM. Medical hypnosis for temporomandibular disorders: treatment efficacy and medical utilization outcome. Oral Surgery, Oral Medicine, Oral Pathology, Oral Radiology, and Endodontology. 2000; 90: 54–63. 10.1067/moe.2000.106692 10884636

[31] Winocur E, Gavish A, Emodi-Perlman A, Halachmi M, Eli I. Hypnorelaxation as treatment for myofascial pain disorder: a comparative study. Oral Surgery, Oral Medicine, Oral Pathology, Oral Radiology, and Endodontology. 2002; 93: 429–434. 10.1067/moe.2002.122587 12029281

[32] Wahlund K, List T, Larsson B. Treatment of temporomandibular disorders among adolescents: a comparison between occlusal appliance, relaxation training, and brief information. Acta Odontologica Scandinavica. 2003; 61: 203–211. 10.1080/00016350310003891 14582587

[33] Abrahamsen R, Zachariae R, Svensson P. Effect of hypnosis on oral function and psychological factors in temporomandibular disorders patients. Journal of Oral Rehabilitation. 2009; 36: 556–570. 10.1111/j.1365-2842.2009.01974.x 19604319

[34] Abrahamsen R, Baad-Hansen L, Zachariae R, Svensson P. Effect of hypnosis on pain and blink reflexes in patients with painful temporomandibular disorders. Clinical Journal of Pain. 2011; 27: 344–351. 10.1097/AJP.0b013e3181ffbfcb 21178599

[35] Ferrando M, Galdón MJ, Durá E, Andreu Y, Jiménez Y, Poveda R. Enhancing the efficacy of treatment for temporomandibular patients with muscular diagnosis through cognitive-behavioral intervention, including hypnosis: a randomized study. Oral Surgery, Oral Medicine, Oral Pathology, and Oral Radiology. 2012; 113: 81–89. 10.1016/j.tripleo.2011.08.020 22669067

[36] Wahlund K, Nilsson IM, Larsson B. Treating temporomandibular disorders in adolescents: a randomized, controlled, sequential comparison of relaxation training and occlusal appliance therapy. Journal of Oral & Facial Pain and Headache. 2015; 29: 41–50. 10.11607/ofph.1285 25635959

[37] Ferendiuk E, Biegańska JM, Kazana P, Pihut M. Progressive muscle relaxation according to Jacobson in treatment of the patients with temporomandibular joint disorders. Folia Medica Cracoviensia. 2019; 59: 113–122. 31891364

[38] Huhtela OS, Koivisto N, Hägg V, Sipilä K. Effectiveness of applied relaxation method *vs.* splint in treatment of temporomandibular disorders in Finnish students. Journal of Oral Rehabilitation. 2020; 47: 123–131. 10.1111/joor.12884 31493297

[39] Ohrbach R, Dworkin SF. The evolution of TMD diagnosis: past, present, future. Journal of Dental Research. 2016; 95: 1093–1101. 10.1177/0022034516653922 PMC5004241 27313164

[40] Ohrbach R, Dworkin SF. AAPT diagnostic criteria for chronic painful temporomandibular disorders. Journal of Pain. 2019; 20: 1276–1292. 10.1016/j.jpain.2019.04.003 31004786

[41] Jessri M, Sultan AS, Tavares T, Schug S. Central mechanisms of pain in orofacial pain patients: implications for management. Journal of Oral Pathology & Medicine. 2020; 49: 476–483. 10.1111/jop.13062 32539196

[42] Ruiz-Marrara J, Antunes LG, Bataglion C, Fernandes MH, Melchior MO, Magri LV. Cognitive domain impairments in chronic painful temporomandibular disorders: associations with pain intensity, hypervigilance and catastrophising: a cross-sectional analysis. Journal of Oral Rehabilitation. 2026; 53: 76–88. 10.1111/joor.70064 PMC12705290 40990683

[43] Schulz-Stübner S, Krings T, Meister IG, Rex S, Thron A, Rossaint R. Clinical hypnosis modulates functional magnetic resonance imaging signal intensities and pain perception in a thermal stimulation paradigm. Regional Anesthesia & Pain Medicine. 2004; 29: 549–556. 10.1016/j.rapm.2004.09.002 15635514

[44] Ambron R. Toward the unknown: consciousness and pain. Neuroscience of Consciousness. 2023; 2023: niad002. 10.1093/nc/niad002 PMC9940454 36814785

[45] Thompson T, Terhune DB, Oram C, Sharangparni J, Rouf R, Solmi M, *et al*. The effectiveness of hypnosis for pain relief: a systematic review and meta-analysis of 85 controlled experimental trials. Neuroscience & Biobehavioral Reviews. 2019; 99: 298–310. 10.1016/j.neubiorev.2019.02.013 30790634

[46] Wahlund K, Larsson B. Long-term treatment outcome for adolescents with temporomandibular pain. Acta Odontologica Scandinavica. 2018; 76: 153–160. 10.1080/00016357.2017.1394490 29073802

[47] Jensen MP, Patterson DR. Hypnotic approaches for chronic pain management: clinical implications of recent research findings. American Psychologist. 2014; 69: 167–177. 10.1037/a0035644 PMC4465776 24547802

[48] Bicego A, Rousseaux F, Faymonville ME, Nyssen AS, Vanhaudenhuyse A. Neurophysiology of hypnosis in chronic pain: a review of recent literature. American Journal of Clinical Hypnosis. 2022; 64: 62–80. 10.1080/00029157.2020.1869517 34748463

[49] Mélou C, Sixou JL, Sinquin C, Chauvel-Lebret D. Temporomandibular disorders in children and adolescents: a review. Archives de Pédiatrie. 2023; 30: 335–342. 10.1016/j.arcped.2023.03.005 37147156

